# Evaluation of the effect of gallic acid dentin treatment on the shear bond strength of the composite resin by using universal adhesive, in self-etch and total-etch strategies

**DOI:** 10.4317/jced.62882

**Published:** 2025-07-01

**Authors:** Saeedreza Ghanbarian, Farahnaz Sharafeddin, Hajar Farhadpour

**Affiliations:** 1 Department of Operative Dentistry, School of Dentistry, Shiraz University of Medical Sciences, Shiraz, Iran; 2 Department of Operative Dentistry, Biomaterials Research Center, School of Dentistry, Shiraz University of Medical Sciences, Shiraz, Iran

## Abstract

**Background:**

Gallic acid (GA), as a material with potentially beneficial effects in dentistry, was used in this study to investigate whether it affects the shear bond strength of composite to the dentin via self-etch and total-etch strategies of a universal adhesive.

**Material and Methods:**

Sixty intact molars with sectioned flat occlusal surfaces were treated with 1 wt.% and 2 wt.% GA solution before applying the universal adhesive in self-etch and total-etch modes. They were randomly divided into six groups (n=10): 1) self-etch control, 2) total-etch control, 3) 1% GA + self-etch, 4) 2% GA + self-etch, 5) 1% GA + total-etch, and 6) 2% GA + total-etch. Teflon molds were fixed on the prepared dentin surface, and then the composite was inserted into the molds and light cured. After spending 24 hours in a storage condition with 100% humidity at room temperature, specimens underwent the SBS test. The SBS values calculated by the universal testing machine were analyzed using ANOVA and Tukey’s post hoc tests (*p*< 0.05).

**Results:**

The application of 1% and 2% GA before using the universal adhesive revealed a significant increase in the mean SBS values compared to respective control groups (*p*< 0.001, *p*< 0.001), and the mean values for 1% GA were significantly higher than 2% GA in both etching strategies (*p*< 0.001, *p*< 0.001). The groups that utilized the universal adhesive with the total-etch method exhibited statistically higher SBS values than those using the self-etch approach, irrespective of applying GA and its concentration (*p*< 0.001).

**Conclusions:**

Applying 1% and 2% GA improved the shear bond strength of composite resin to the dentin in both self-etch and total-etch approaches with the universal adhesive. These findings indicate that GA holds great potential for expanded clinical applications.

** Key words:**Gallic acid, shear bond strength, universal adhesive.

## Introduction

A sTable, high-quality hybrid layer is essential for a durable bonding interface, relying on the interaction between resin monomers and collagen fibrils for micromechanical interlocking (1). The hydrated nature of the dentin makes proper resin infiltration challenging, leaving some collagen fibrils unprotected, resulting in bonding failure and reduced durability (2,3).

Universal adhesives, the eighth generation of adhesive systems, are designed to minimize the application steps and reduce the technique sensitivity. They can be used on different moisture levels of the enamel and dentin in total-etch, self-etch, and selective etch modes. Various studies have examined how different application modes affect the nanoleakage and bond strength of these adhesives (4,5).

According to previous studies, collagen cross-linking agents (CCLAs) can be effective in stabilizing the hybrid layer and improving the mechanical properties of the dentine substrate, and several strategies have been introduced over time (6). Natural cross-linkers like proanthocyanidins (PACs), riboflavin, and epigallocatechin-3-gallate (EGCG) seem more appealing than the other synthetic materials, so they reduce potential cytotoxic issues. For instance, riboflavin pretreatment could potentially increase the bond strength and participate in achieving a more durable hybrid layer (7). CCLAs can be applied in various methods like dentin pretreatment as a separate step, mixing into the adhesive system, or adding to the etchant (8). These materials can stiffen the collagen network and enhance some mechanical properties of the dentin substrate, which can result in better bonding strength (9).

 GA is classified as a type of PAC trimer and is highly water-soluble, making it easily absorbed in biological systems (10). This compound is commonly found in various natural sources, most notably in grapes and green tea. GA exhibits a range of biological effects that contribute to its significance in health and medicine. Notably, it demonstrates powerful anti-inflammatory properties, which can help reduce inflammation in various tissues (11). Additionally, GA has shown to be effective as an antiviral agent, working to inhibit the replication of certain viruses (12). Furthermore, its antibacterial activity is particularly noteworthy, as it effectively targets cariogenic bacteria, the microorganisms responsible for dental caries (13). Previous studies have also highlighted additional properties of GA and its derivatives in the dental field. These properties include the ability of free radical scavenging and act as an antioxidant, Ca2+ chelator activity, induction of mineralization capacity, and enamel anti-demineralization effect (14-16). Furthermore, it has been observed that GA can imitate the natural cross-linking processes that occur within the dentin. This imitation of cross-linking mechanisms contributes to a significant reduction in the biological degradation rate of collagen fibers found in the dentin matrix. As a result, the structural integrity and longevity of collagen are enhanced, which may lead to improved outcomes in dental treatments and restoration procedures (17). It has been shown that GA derivatives, such as Catechin Gallates and Gallocatechins, play a role in inhibiting the function of matrix metalloproteinases (MMPs) and cysteine cathepsins. The inhibitory effects of these compounds on MMPs and cathepsins suggest that Catechin Gallates and Gallocatechins could be valuable in therapeutic strategies which aim at managing conditions where excessive matrix degradation is a concern (18).

The shear bond test (SBS) is a simple in-vitro method for evaluating the effectiveness of dental adhesives in bonding them to the substrate. Its advantages include easy specimen preparation and relevance to chewing forces, which is the reason for employing the SBS test in this study.

In the current in-vitro study, we developed an experimental protocol by using 1% and 2% GA solution before applying Gluma Bond Universal (GBU) in self-etch and total-etch strategies to evaluate the effect of GA on the shear bond strength of composite resin to the dentin. The null hypothesis was: The shear bond strength of the composite to the dentin increases after applying 1% and 2% GA solution in the both self-etch and total-etch strategies.

## Material and Methods

**Figure 1:**
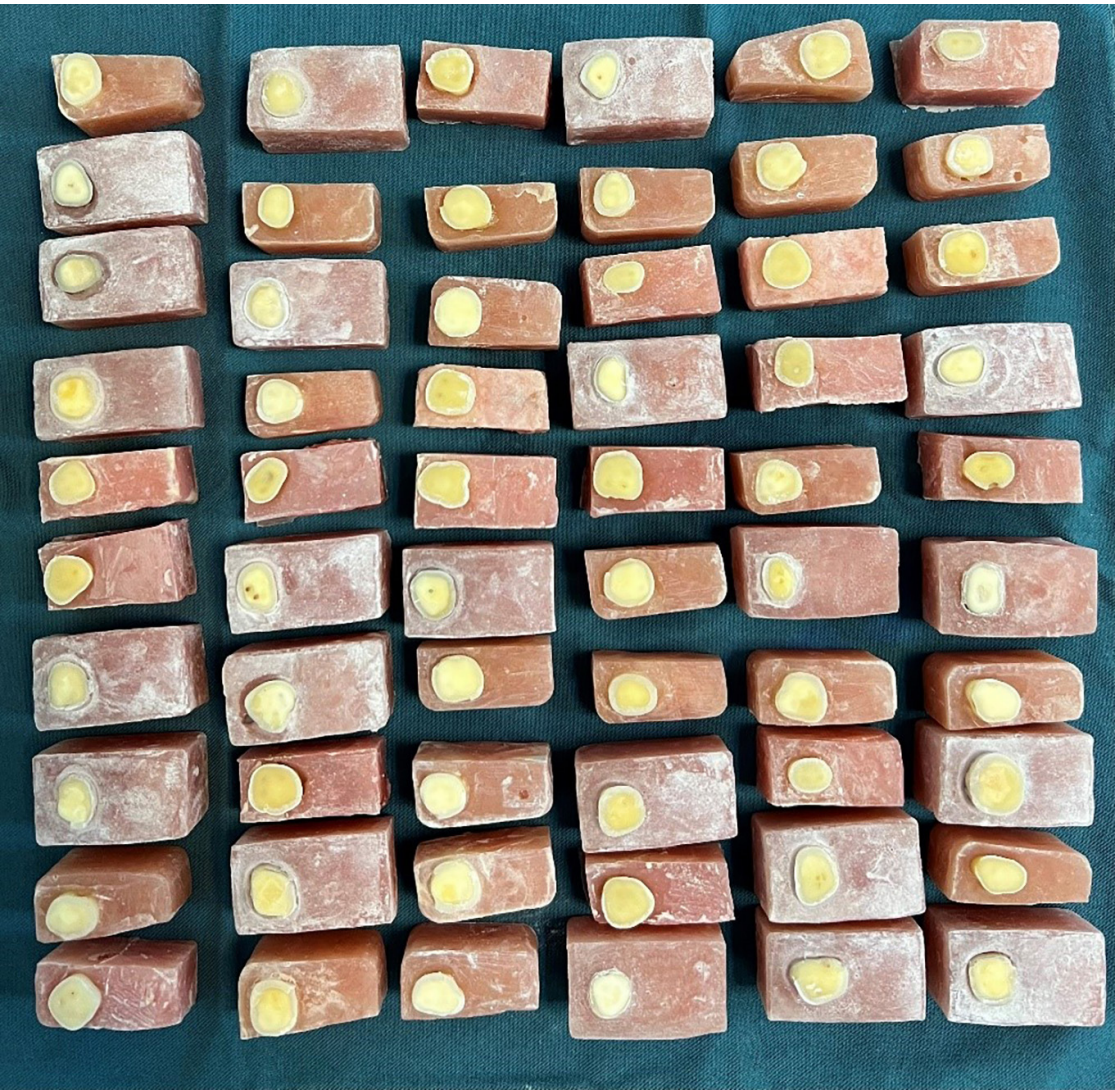
Prepared specimens.

The ethics committee of the university approved the present experimental study with the code of IR.SUMS.DENTAL.REC.1402.089. An overall count of 60 human molar teeth without caries and previous restoration and fracture were considered for the study, which was collected for two months before the start of the research and washed until the debris was completely cleaned. All the teeth were removed for orthodontic or periodontal issues with the patients’ informed consent at the discretion of another dentist who was not participating in this study, and the implementers of this project were not involved in the tooth extraction process. The teeth were kept in a 0.1% thymol (Merck, Darmstadt, Germany) solution in a refrigerator at 4°C. Then, they were washed under high-pressure water. Then, self-curing acrylic resin molds (Acropars, Iran) with dimensions of 1.5 × 1.5 × 3 cm were prepared, and the teeth were mounted during the acrylic resin setting so that their occlusal surface was parallel to the upper surface of the acrylic resin. The coronal surface of the embedded teeth was sectioned transversally with a diamond disc (D & Z, Germany) to expose the dentin at a depth of 2 mm below the DEJ; then, the final finishing procedure was done with a standard 600-grit silicon carbide paper to obtain a smooth surface (19) (Fig. 1). Six groups were formed by taking a random selection of samples (n=10).

Group 1: Gluma Bond Universal (GBU), as a bonding agent, was applied with active rubbing motion on the dentin surface by a microbrush (Premium Plus International Ltd., Hong Kong) in 2 alternating layers for 20 seconds as self-etching mode. To ensure the solvent evaporation, the surface was air-dried with a gentle flow of air until the adhesive film no longer moved, and then the LED light curing device (BlueLEX, Monitex, Taiwan) was used for 10 seconds, featuring an intensity of 1200 mW/cm² and operating at a wavelength of 450 nm. Group 2: The teeth were etched with 37% phosphoric acid )Condac37, FGM, Brazil) for 15 seconds, rinsed for 20 seconds, and gently air-dried. Then, the same procedure of bonding application for the previous group was followed.

The prepared GA solution was used in the next groups. After scaling the GA powder, 1% wt. and 2% wt. GA solutions were prepared by dissolving 1 gr and 2 gr of GA powder (Gallic acid, Sigma Aldrich, USA) in 100 ml of distilled water. Group 3: The prepared 1% GA solution was applied for 10 seconds, rubbed by a microbrush, and then rinsed with water for 10 seconds (20). Afterward, GBU was used as a self-etch mode in 2 alternating layers and then light cured for 10 seconds. Group 4: In this group, the teeth were rubbed with a 2% GA solution for 10 seconds by a micro brush, and then the following procedure was done similarly to the previous group. Group 5: After applying 37% phosphoric acid for 15 seconds and then rinsing for 15 seconds, 1% GA solution was applied on the dentin surface for 10 seconds with a microbrush; then, flushed out with water for 10 seconds, and then GBU was applied in 2 layers for 20 seconds as a total-etch mode, and light cured for 10 seconds. Group 6: In this group the same total-etch procedure was done, with the difference of applying 2% GA solution on the teeth. Also, the following procedure was done like group 5.

After preparing the surface of the dentin by the mentioned methods, the micro-hybrid composite (Shade A2, Charisma Smart, Kulzer, Germany) was placed using a Teflon mold measuring 3 mm (diameter) × 2 mm (height) and then light cured for 40 seconds from the top. Experimental materials used in this article and their characteristics are presented in  Table 1.

**Figure 2:**
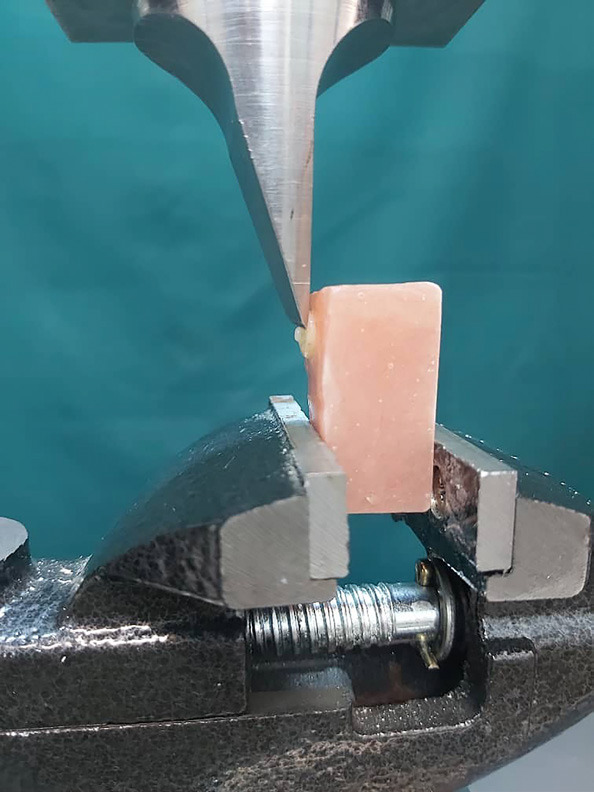
The mounted specimen for the SBS test.

 All specimens were stored under 100% humidity for 24 hours at room temperature. The prepared samples were carefully positioned in the universal testing machine (Instron Z020, Zwick/Roell, Germany) to ensure proper alignment and stability during testing. A shear force was then systematically applied to each sample using a knife-edge blade, which ensured a focused application of force (Fig. 2). The crosshead was set to move at a controlled speed of 1 mm/ min, allowing for gradual loading until the samples reached their failure point. Throughout this process, the force values (N) were continuously recorded. Following the completion of the test, the SBS of each sample was calculated and presented in MPa, allowing for a comprehensive analysis of the material’s performance under shear stress.

The tested samples underwent a thorough examination using a stereomicroscope (BestScope; BS-3060C) set at a magnification of ×40 to accurately identify the various types of failure occurring within the samples. The observed failure modes were categorized into four different types.

1. Cohesive failure in the dentin: Indicating that the bond strength is sufficient at the interface but insufficient within the dentin structure.

2. Cohesive failure in the composite: Indicating a loss of cohesion among the particles or phases of the composite resin, rather than at the bonding site.

3. Adhesive failure at the interface: This failure mode involves a breakdown at the junction where the composite material bonds with the dentin surface.

4. Mixed Failure (Cohesive and Adhesive): This category encompasses instances where cohesive (within the composite or dentin) and adhesive failure occur simultaneously.

SPSS version 26 (SPSS Inc., USA) was used for the statistical analyses. The values for the SBS were reported as the mean plus their respective standard deviation (SD). To evaluate the normality of the data, we employed the Shapiro-Wilk test. Subsequently, a two-way ANOVA was conducted for data analysis. To perform post hoc comparisons of the means among the different groups, we used the Tukey test after an analysis of variance. Additionally, the evaluation of failure modes across the various study groups was conducted using Fisher’s exact test, to determine if there are nonrandom associations between categorical variables. (*P* value <0.05 ).

G-Power software (G*Power 3.1 software; Heinrich Hein University, Dusseldorf, Germany) was used for power analysis which was conducted after the statistical analysis showed that the power level of the test based on effect size for the variable of the concentration of GA (f = 1.13) and for the variable of etching type (f = 0.731) at type I error (α) of 0.05 level of was 99.9% and 97%, respectively, which was higher than the offered power level of 80%.

## Results

**Figure 3:**
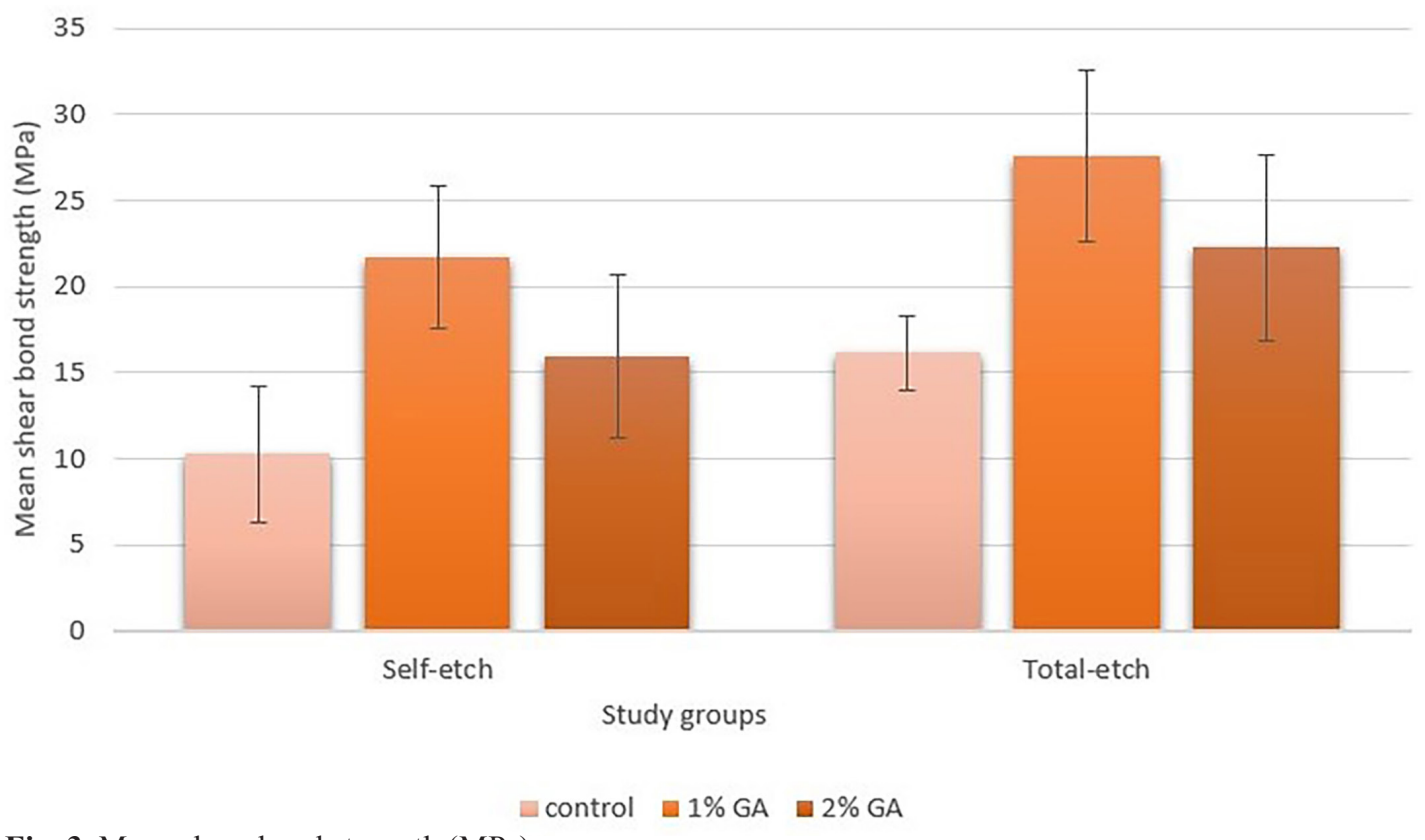
Mean shear bond strength (MPa).

 Table 2 and Figure 3 present the mean SBS and standard deviations for the study groups. Two-way ANOVA and subgroup analysis were employed to compare the mean SBS of the study groups. SBS was significantly influenced by applying 1% and 2% GA and the etch type (*p* < 0.001).

Post hoc Tukey’s tests were used for pairwise comparisons between materials. The results revealed that the mean SBS value in group 5 (total-etch + 1% GA) was significantly the highest value among the study groups (*p* < 0.001). Statistically higher SBS values were observed when 1% GA was applied compared to 2% GA in self-etch and total-etch strategies (*p* < 0.001, *p* < 0.001). On the other hand, pretreatment with 2% GA in both etch strategies resulted in significantly higher values than their corresponding control groups (*p* < 0.001, *p* < 0.001). Applying 1% and 2% GA after the total-etch strategy resulted in significantly higher mean SBS values compared to the self-etch strategy with the same concentration of GA (*p* < 0.001).

**Figure 4:**
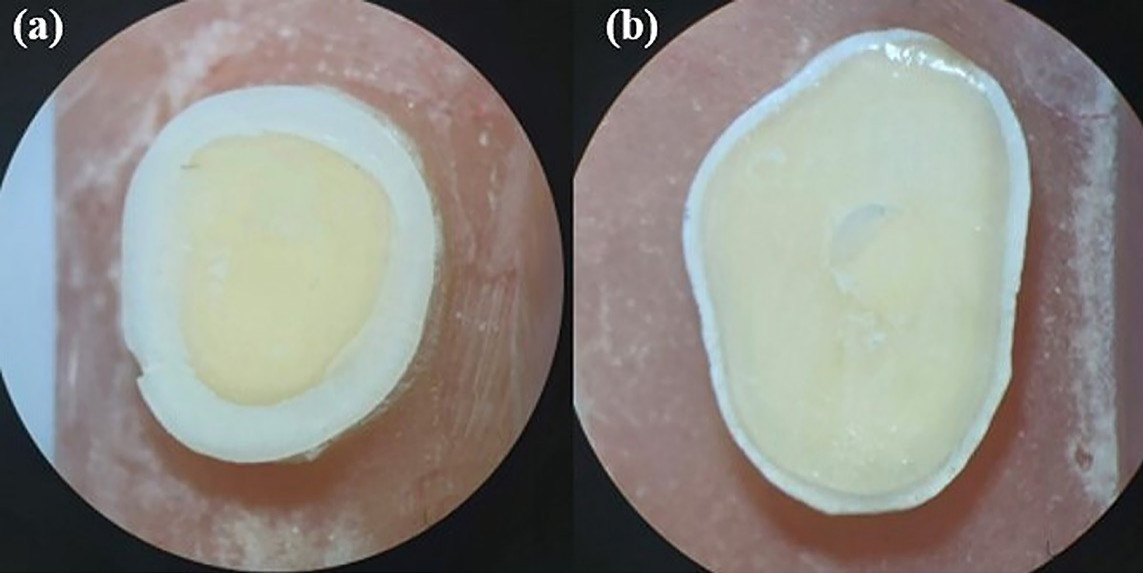
Observed failure modes: Adhesive (a), mixed (b).

The prevalence of the failure modes is presented in  Table 3 for each group. The failure analysis indicated that in group 1, the sole type of observed failure type was adhesive. In contrast, adhesive and mixed failure modes were observed in all the other experimental groups. Note that cohesive failure in the dentin and composite materials was not observed in any of the experimental groups (Fig. 4). The results of Fisher’s exact test indicated no statistically significant difference between the tested groups (*p* = 0.203).

## Discussion

It has been reported that plant extracts containing proanthocyanidin (PAC) can effectively enhance the biomechanical properties, integrity, and stability of resin-dentin bonds by acting as natural cross-linking agents, and one such example is GA (21). The findings indicated that applying 1% and 2% GA significantly enhanced the SBS of composite resin to the dentin, especially within the total-etch approach. Consequently, the null hypothesis is considered acceptable.

Goncu *et al*. (20) focused on examining the SBS of Single Bond Universal adhesive (SUA) on contaminated and non-contaminated dentin using three different application protocols. These protocols included pre-conditioning with either 35% phosphoric acid or 1% GA or without any pre-conditioning step. The researchers found that the highest SBS values among different treatments were obtained with the GA dentin pre-conditioning undergoing different contaminations. Through SEM (Scanning electron microscope) analysis, the researchers noted that the surface of the sample treated with 1% GA showed predominantly semi-open dentinal tubules with a crack-like appearance and fully open dentinal tubules. As a result, they concluded that 1% GA was probably effective in eliminating contaminants without overly demineralizing the dentin. These results are in the same line with our findings, where we observed that pretreatment with 1% and 2% GA groups showed better results than the control groups. However, unlike SUA, applying phosphoric acid before using GBU resulted in higher SBS values compared with self-etch mode. This phenomenon seems to be due to the properties of each universal bonding like their acidity, containing functional monomers, and the application protocol which depends on the manufacturer’s instructions for use.

In a study by Vidal *et al*. (22), various compounds including PACs, GA, its derivatives, and a glucose ester were assessed for their effect on the dentin modulus of elasticity via flexural test. In their study, dentin substrates were demineralized by 10% phosphoric acid for 5 hours to expose the organic matrix. They found that among GA and its derivatives, only penta-galloyl-glucose (PGG) improved the mechanical properties of the dentin matrix. Additionally, they stated that, in compounds like GA, the molecular weight significantly influenced their interaction with the dentin matrix. Consequently, GA had a restricted capacity for cross-linking dentinal collagen fibers. These results are somehow in line with those of the present study. Methods for demineralizing the dentin and its extent might be different; however, the reciprocal effect of adhesive and GA might play a role in obtaining better results in our study. As we observed, applying 1% and 2% GA significantly increased the mean SBS.

Universal adhesives, due to their acidic functional monomer content, can benefit from both self-etching and total-etching strategies for substrate demineralization, resulting in varying degrees of acidic pH. According to previous studies, controversial results about the effectiveness of applying phosphoric acid before the bonding agent were observed (23,24). According to the results of this study, total-etch groups demonstrated significantly higher SBS, irrespective of the GA concentration. The discrepancy among the groups might be attributed to different protocols used for testing the bond strength and different application procedures.

The different application modes of the universal adhesives affect the surface morphology and the resin penetration along the exposed collagen fibrils which can affect the resin/dentin bond strength (24). As mentioned before, in our study, GBU showed statistically higher bonding strength in total-etch mode compared to self-etch mode regardless of different surface pre-treatments. GBU contains 10-MDP which is an acidic functional monomer that chemically interacts with hydroxyapatite (25). Therefore, GBU is very acidic (pH=1.5) and is categorized as an intermediately strong universal adhesive due to acidity (26). On the other hand, applying 37% phosphoric acid gel before bonding application causes greater demineralization than in self-etch mode. It was assumed that applying 37% phosphoric acid can increase the free surface energy, making a substrate more suitable for the adhering mechanism of GBU. Also, one might expect that treating the surface with GA besides phosphoric acid causes a higher value of demineralization, and utilizing these three acidic agents on the dentin substrate would lead to lower SBS than the control self-etch group, but other factors are important as well. Factors like acid dissociation constant (pKa), molecular weight, solution concentration, and application duration are involved in determining the extent of dentin demineralization. In addition, one of the beneficial aspects of GA is its potential effect in enhancing remineralization and inhibiting demineralization due to previous studies (27). This capability seems to limit the dentin demineralization and creates higher bonding performance than the control groups. Thus, the total-etch group with the 1% GA showed significantly higher values than the other groups, and the self-etch control group without any pre-treatment showed the lowest value.

Exposing the dentin tissue to acidic conditions such as phosphoric acid in the total-etch system and acidic functional monomers such as 10-MDP in self-etch systems activates the host-derived enzymes. These enzymes, including MMPs and cysteine cathepsins, break down the exposed collagen fibrils and participate in bond degradation over time. In total-etch adhesive systems, poorly resin infiltration through a demineralized collagen matrix is more common, leaving collagen fibrils unprotected (28). However, due to milder substrate etching by self-etch adhesives, this phenomenon occurs to a limited extent. In a study by Bedir *et al*. (24), different MMP inhibitors, including EGCG, the ester of epigallocatechin and GA, were used and the EGCG groups showed promising results. Besides existing cross-linking capacity, the MMP inhibitory effect of GA has been demonstrated in a previous study (22), where significantly lower collagen degradation was observed for GA, and the reason for this was related to its capability to attach to collagen molecules at or close to the points where they separate as “cleavage sites”. This capacity may have contributed to achieving higher SBS values for 1% and 2% GA pretreatment compared to the respective control groups; however, this feature affects the bond durability more than its immediate bond strength.

Following the acid-etching process, it is crucial to consider the effects of prolonged exposure of the dentin tissue to high airflow. Such exposure can lead to a significant loss of water content surrounding the collagen fibrils, ultimately resulting in their structural collapse. When this collapse occurs, it has a detrimental impact on the bond strength and durability of the hybrid layer (29). Materials like GLUMA desensitizer have been introduced as rewetting agents and can effectively restore the collapsed fibrils to near their original state (30). In our study, after applying phosphoric acid and subsequently drying the dentin surface, the application of 1% and 2% GA showed promising results in increasing the SBS compared to the control group. However, this improvement may be attributed to the presence of water in the GA solution and may help to expand the interfibrillar space thereby facilitating better interaction and bonding with the adhesive. Hence, the findings emphasize the importance of managing moisture levels during bonding procedures to enhance the effectiveness and durability of dental restorations.

Free radicals are an essential part of the process of resin polymerization, irrespective of the activation type. These free radicals can result from energy (heat or light) or chemical activation. GA and its derivatives can prevent oxidation because of high oxygen-derived free radical scavenging activity due to polyphenolic functionality (31). The higher the concentration of GA in the bonding area, the greater the disruption to resin polymerization through the increased free radical scavenging effect. Hence, incorporating GA into the adhesive seems to be out of benefit and even interrupts the monomer conversion and bond strength. In a previous study, de Souza *et al*. (32) suggested that the incorporation of proanthocyanidins, such as GA, into the adhesive resin can have detrimental effects on the adhesive interface. These compounds may interfere with the polymerization process by inhibiting both the initiation phase, where polymer chains begin to form, and the propagation phase, where these chains grow and solidify. On the other hand, Neri *et al*. (33) found that merging 0.1 % and 0.01% EGCG with a self-etch bonding agent did not have any negative effect on the degree of conversion or flexural strength values; consequently, the concentration of the material for this phenomenon matters. In the present study, a higher concentration of GA molecules led to weaker results of SBS compared to a lower one, which seems to be due to the increasing impact of its free radical scavenging effect; however, in 1% GA more positive features are predominant. Still, applying 2% GA showed better results than the corresponding control groups.

Prajatelistia *et al*. (16) stated that one of the aspects of GA is the chelating ability to create metal ion complexes like GA/Fe3+ which causes dentinal sealing and relieves dentin hypersensitivity. The main mechanism of this complexation was related to the potency of adhering and cross-linking on the surface of dentinal tubules and creating new nucleation sites of hydroxyapatite. In their study, over analysis, the observations under SEM revealed that the GA/Fe3+ complex showed the highest occlusion of the dentinal tubules among all the complexes. In the present study, pre-treatment with 2% GA showed weaker results than 1% GA pre-treatment. As an assumption, higher concentrations of GA might interfere with resin infiltration to the dentin substrate by forming metal ion complexes, especially in self-etch mode due to a higher amount of deposit minerals in the smear layer, which could quickly and efficiently seal the tubules.

By classifying the failure modes, a comprehensive understanding of the types of failures encountered can be achieved, which is essential for improving material performance and bonding techniques. In this study, the most common type of bond failure in all groups was found to be adhesive failure, as we see in some previous similar studies (20). Although the cohesive failures were not observed in any group, neither in the dentin nor in the composite, mixed failures were found in all groups except the self-etch control group. In a meta-analytical study (34), a relationship was observed between the prevalence of different failure modes and the mean bond strength. As a result, higher mixed failures and lower adhesive failures were seen in the group with the highest mean SBS value, which was the total-etch group with 1% GA pre-treatment. On the other hand, the control self-etch group only showed adhesive failure which seems to be due to the lower SBS among the tested groups. In the present study, cohesive failure was not observed in any group, and adhesive failure was the most common one. Consequently, it seems that, as before, the adhesive interface is the weak point of the dentin/composite complex and most failures occurred here. Furthermore, there was no significant difference between the groups in the failure mode statistical analysis; however, applying 1% GA resulted in a lower occurrence of adhesive failure in comparison with other groups.

Further studies are needed to investigate the differences between GA and its derivatives in the various concentrations and their effects on the resin/dentin interface and mechanical properties. Complementary *in-vivo* studies should be performed to evaluate the effect of GA in clinical situations. Also, in this study, only one universal adhesive was utilized, and the effect of GA on other adhesives should be evaluated in future studies. Other types of substrates with different contamination factors that might affect the bond strength should be evaluated in future studies.

## Conclusions

Recent research indicates that GA, as an emerging material in dentistry, can significantly enhance the microshear bond strength between composite resins and dentin, which is crucial for the longevity and effectiveness of dental restorations. Notably, this study reveals that the most pronounced increase in bond strength occurs when GA is applied at a concentration of 1%.

## Figures and Tables

**Table 1:** Experimental materials and their characteristics.

Material	Composition	Manufacturer	Lot no.
Gluma Bond Universal	4-META, UDMA, Acetone, MDP, water	Heraeus Kulzer GmbH, Hanau, Germany	M010060
Charisma Smart (A2)	Bis GMA, TEGDMA, Barium Aluminium Fluoride glass, silicon dioxide, 59% filler by volume	Heraeus Kulzer GmbH, Hanau, Germany	M010543
Gallic acid	3,4,5 Trihydroxybenzoic acid (C7H6O5)	Sigma-Aldrich Chemie, Steinheim, Germany	S8162349525
Condac 37	37% phosphoric acid gel, dye, deionized water, thickener	FGM, Joinville, Brazil	040822

**Table 2:** Means and standard deviations (SD) for SBS in the tested groups (MPa). Note: The mean values of the different letters were statistically significant by post hoc Tukey’s test (p < 0.05).

	Control (mean ± SD)	1% GA (mean ± SD)	2% GA (mean ± SD)	p Value*
Self-etch	10.27 ± 3.96A	21.72 ± 4.12C	15.93 ± 4.73B	<0.001
Total-etch	16.16 ± 2.18B	27.59± 4.94D	22.28 ± 5.39C	<0.001
P value	<0.001	<0.001	<0.001	-

GA: gallic acid

**Table 3:** Type and prevalence of the failure modes in each experimental group (n=10).

Experimental condition	Cohesive in dentin	Cohesive in composite	Adhesive	Mixed
Control (Self-etch)	0	0	10	0
Control (Total-etch)	0	0	8	2
Self-etch + 1% GA	0	0	7	3
Self-etch + 2% GA	0	0	7	3
Total-etch + 1% GA	0	0	5	5
Total-etch + 2% GA	0	0	7	3

GA: gallic acid

## Data Availability

The data that support the findings of this study are available in the main text.
